# Development of a nomogram model to predict survival outcomes in patients with primary hepatic neuroendocrine tumors based on SEER database

**DOI:** 10.1186/s12885-021-08337-y

**Published:** 2021-05-18

**Authors:** Ziteng Zhang, Xin Zhao, Zhiyan Li, Youchun Wu, Yao Liu, Zhiwei Li, Guobao Li

**Affiliations:** 1 Department of Hepato-Biliary Surgery, Shenzhen Third People’s Hospital, The Second Affiliated Hospital, Southern University of Science and Technology, Shenzhen, 518055 China; 2 Department of Ultrasonography, Shenzhen Third People’s Hospital, The Second Affiliated Hospital, Southern University of Science and Technology, Shenzhen, 518055 China; 3 Department of Hepatology, Beijing Hospital of Traditional Chinese Medicine, Capital Medical University, Beijing, China; 4 Department of Lung Disease, Shenzhen Third People’s Hospital, The Second Affiliated Hospital, Southern University of Science and Technology, Shenzhen, 518055 China

**Keywords:** Primary hepatic neuroendocrine tumors (PH-NETs), Nomogram model, SEER database

## Abstract

**Background:**

Primary hepatic neuroendocrine tumors (PH-NETs) are extremely rare and unknown. Because of its rarity, its prognosis features and influencing factors are not well established.

**Methods:**

Data of 140 patients with PH-NETs diagnosed in the SEER database from 1975 to 2016 were collected. The demographics and clinic-pathological features were described. By using propensity-score matching (PSM) analysis, three associated cohorts were selected to describe the malignancy of PH-NETs and univariate analysis was conducted. Then, multivariate Cox analyses were performed and a predicting nomograph was constructed. C-index, receiver operating characteristic (ROC) curve and calibration curves were used to evaluate the predictive value of nomogram.

**Results:**

The overall survival outcomes of PH-NETs were superior to hepatocellular carcinoma (HCC) with a mean survival time 30.64 vs 25.11 months (*p* = 0.052), but inferior to gastrointestinal tract neuroendocrine tumors in situ (GI-NETs in situ) with a mean survival time 30.64 vs 41.62 months (*p* = 0.017). With reference to gastrointestinal neuroendocrine tumors with liver metastasis (GI-NETs-LM), GI-NETs-LM had better outcomes in short time (1-year survival rate: 64.75% vs 56.43%) but was worse in long time (5-year survival rate: 8. 63% vs 18.57%). Multivariate Cox analyses showed that tumor grade and surgery were two independent factors for prognosis of the patients (*p* < 0.00). Tumor grade and surgery were used to construct the predicting nomogram. The C-index was 0.79 (95%CI = 0.75–0.83). The area under curve (AUC) values in ROC were 0.868 in 1-year and 0.917 in 3-year survival and the calibration curves showed good consistency.

**Conclusions:**

The overall prognosis PH-NETs is generally favorable, better than HCC and GI-NETs-LM in long term. Preoperative biopsy and complete pathological diagnosis were recommended. Radical surgical intervention including transplantation was the first choice in PH-NETs therapy.

**Supplementary Information:**

The online version contains supplementary material available at 10.1186/s12885-021-08337-y.

## Background

Neuroendocrine tumors (NETs) cover a wide range of neoplasms that originate in the neuroendocrine cells that spread throughout the body. Recent study have suggested a 6.4-fold increase in its incidence rate from 1973 (1.09 per 100,000) to 2012 (6.98 per 100,000) [1, 2]. According to a recent United States population-based studies, gastrointestinal tract (GI) was the most frequently occurring site in NETs, followed by lung. Within the GI tract, rectum and small intestine accounted for over half of NETs prevalence [2]. Primary hepatic neuroendocrine tumors (PH-NETs) are rare neuroendocrine tumors, representing 0.4% of all NETs, and were first described by Edmonson in 1958 [3, 4].

Hepatocellular carcinoma (HCC) and cholangiocarcinoma (CC) were the first two most common primary malignant hepatic tumors (PMHTs). Other than them, the most common PMHTs were neuroendocrine tumor and lymphoma which were infrequent and poorly known [5].

The origin of PH-NETs remains unclear. Three possible theories have been proposed for the origin which were possible transformation of hepatocellular carcinoma with carcinoid features [6]; malignant transformation from the neuroendocrine cells scattered among the intrahepatic biliary epithelium [7, 8]; ectopic heterotopic pancreatic or adrenal tissue in the liver [9]. However, none of them has been confirmed.

Diagnosis of PH-NETs requires both histopathological confirmation and the absence of extrahepatic primary sites. Pathological examination could be achieved by fine needle aspiration or biopsy. Microscopically, NETs exhibit characteristic features, including trabecular or insular architecture, peripheral palisading of cells, abundant vascularity of the stroma, uniform cell size and generally rounded nuclei [10]. Immunohistochemistry was routinely operated by detecting potential markers such as CgA (chromogranin A), NSE (neuron-specific enolase), CST (chromostatin), CEA (carcinoembryonic antigen) or SYP (synaptophysin) and might indicate the origin of PH-NETs [11, 12]. However, histology alone was insufficient to differentiate primary and metastatic liver NETs. Given the fact that liver is the most frequently metastatic organ from GI-NETs, the diagnosis of a PH-NETs must follow demands thorough preoperative examination, operative inspection, and careful follow-up to exclude potential extrahepatic primary sites [13].

Although PH-NETs has long been considered to be a low malignancy with relative slow progression, the clinical features and prognosis results have not been well recognized due to its rarity [14]. In present, most of concerning literatures are case reports or small cohort studies from single institution and reference value is very limited [15–18]. According to them, surgical intervention is considered to be an effective treatment for PH-NETs. Reported patients receiving surgical resection or even liver transplantation have a desirable curative effect and a promising long-term survival rate [13, 19]. Literatures have reported an over 70% 5-year survival rate after surgical treatment and post-resection perihepatic lymph node examination reported infrequently lymph node involvement [10, 20]. With regard to other potential prognostic factor for PH-NETs, such as age, operability, tumor grade and tumor stage, no solid evidence is available at present.

Therefore, the goal of this study was to retrospectively analyze the demographic and clinic-pathological features of PH-NETs patients registered in the Surveillance, Epidemiology and End Results (SEER) database from 1975 to 2016 and more importantly to unveil the prognostic factors influencing PH-NEN survival.

## Methods

### Data source

The Surveillance, Epidemiology, and End Results (SEER) data-base from 1975 to 2016 was used to collect patients’ demographics, clinic-pathological features and survival data. This database also provides additional treatment fields data from 18 population-based cancer registries based on the November 2018 submission, covering approximately 30% of the US population [21].

SEER*Stat software (version 8.3.8; National Cancer Institute, Bethesda, MD, USA) was used to extract information from the database. The SEER program is publicly accessible, which merely contains anonymous patient information. Thus, our study was exempt from the ethical review or the patient consent.

### Study population

In order to enroll primary hepatic neuroendocrine tumors (PH-NETs) patients, the primary site was defined by the following International Classification of Diseases for Oncology (ICD-O-3) codes: liver (C22.0). Histological types were defined by the following ICD-O-3 histology/behavior codes: 8013/3, 8153/3, 8156/3, 8240/3, 8241/3, 8242/3, 8243/3, 8244/3, 8245/3, 8246/3, 8249/3, 8574/3 (variants of neuroendocrine tumors and carcinoids). Patients with incomplete pathological diagnosis information were excluded. Furthermore, in order to exclude the possibility of NETs liver metastasis, patients with distant metastasis were excluded.

In the propensity-score matching (PSM) analysis, we selected three cohorts including common-type hepatocellular carcinoma without metastasis (HCC in situ), gastrointestinal neuroendocrine tumors without metastasis (GI-NETs in situ) and gastrointestinal neuroendocrine tumors with liver metastasis (GI-NETs-LM). HCC in situ were defined by primary site of liver (C22.0) and histological type code of 8170/3. The primary site of GI-NETs includes esophagus (C15.9), stomach (C16.9), small intestine (C17.9), colon (C18.9), rectum (C20.9), and anus (C21.0). The histological types were consistent with the description in PH-NETs.

### Variables collected

The following clinic-pathological variables were used for this study are as follows: patient ID, age at diagnosis, race, gender, insurance recode, marital status, tumor grade, SEER historic stage A (1973–2015), Joint Committee on Cancer (AJCC) 6th TNM stage, tumor size, lymph nodes invasion, distant metastasis, liver metastasis, AFP (alpha fetoprotein), fibrosis score, total tumor number in situ, surgery, chemotherapy, radiation recode and survival months.

### Statistical analysis

The Kaplan-Meier approach was utilized to estimate survival probabilities and a log-rank test was applied to evaluate the difference in survival stratified by each variable. Propensity score matching (PSM) was conducted in 1:1 pattern based on following variables, including age, race, gender, insurance recode, marital status, tumor grade, lymph nodes invasion, tumor size, total tumor number, surgery, chemotherapy and radiation. Multivariate Cox regression was utilized to identify significant variables affecting survival and the survival curve was drawn based on respective variables [22]. The 1-year, 3-year, 5-year survival rate, Hazard Ratio (HR) and 95% CI were calculated.

To predict the prognosis of PH-NENs patients, a nomogram was drawn according to the variables identified in the multivariate Cox analysis. The established nomogram was further evaluated by using concordance index (C-index) and calibration curves [23, 24]. In addition, the precision of the prognosis prediction was evaluated using the area under receiver operating characteristic (ROC) curve (AUC) [25]. Statistical analysis was performed by using SPSS software version 22 (SPSS Inc., Chicago, IL, USA) and GraphPad Prism 7 (GraphPad Software). R language 3.6.1 software was used to determine the prognostic nomogram, C-index, calibration and ROC curve. (The R Foundation for Statistical Computing, Vienna, Austria. http://www.r-project.org).

## Results

### Patient characteristics and univariate analysis

In the SEER database, 635 patients were initially identified from 1975 to 2016. Of them, 345 patients were excluded because of insufficient pathological diagnosis details. Furthermore, 150 patients were excluded due to distant metastasis records. A final total of 140 patients were identified and included in the current study.

The patients’ demographics and clinic-pathological features are depicted in Table 1. Mean survival time stratified by each variate was also calculated (Table 1). The overall mean survival time of PH-NETs patients was 30.64 months (95%CI = 24.75–36.53, Table 2). The overall 1-year, 3-year, and 5-year survival probability was 56.43, 30.00, and 18.57%, respectively (Table 2).

**Table 1:** Patients characteristics and univariate analysis

Variables	Survival time		Survival number and rate						Univariate analysis	
	Number	Mean survival time [95% CI] (months)	1-year survival		3-years survival		5-years survival			
Total number	140		Number	Rate [95%CI]	Number	Rate [95%CI]	Number	Rate [95%CI]	HR [95%CI]	*P*-value
Age (years):										0.002
00–24:	1	16.00	–	–	–	–	–	–	Ref	
25–49:	28	50.75 (35.20–66.30)	22	78.57 (60.46–89.79)	15	53.57 (35.81–70.47)	9	32.14 (17.93–50.66)	1.04 (0.14–7.58)	0.97
50–74:	68	30.33 (21.98–38.68)	36	52.94 (41.24–64.33)	22	32.35 (22.44–44.16)	15	22.06 (13.85–33.26)	0.38 (0.23–0.63)	0.00
> 75:	43	18.05 (10.82–25.28)	20	46.51 (32.51–61.08)	5	11.63 (5.07–24.48)	2	4.65 (1.28–15.46)	0.64 (0.43–0.96)	0.03
Race:										0.20
black:	24	25.96 (15.72–36.20)	15	62.50 (42.71–78.84)	6	25.00 (12.00–44.90)	3	12.50 (4.34–31.00)	Ref	
other:	9	16.78 (0.92–32.63)	4	44.44 (18.88–73.33)	1	11.11 (1.99–43.50)	1	–	1.25 (0.79–1.96)	0.34
white:	107	33.05 (25.82–40.29)	60	56.07 (46.62–65.11)	35	32.71 (24.55–42.06)	22	20.56 (13.99–29.17)	1.73 (0.87–3.43)	0.12
Sex:										0.24
Female:	85	33.29 (25.58–41.01)	49	57.65 (47.04–67.60)	27	31.76 (22.84–42.27)	18	21.18 (13.84–31.01)	Ref	
Male:	55	26.84 (17.73–35.94)	30	54.55 (41.52–66.97)	15	27.27 (17.28–40.23)	8	14.55 (7.56–26.16)	0.82 (0.58–1.15)	0.25
Marital status:										0.78
Married:	75	32.57 (23.89–41.25)	45	60.00 (48.69–70.34)	21	28.00 (19.10–39.04)	14	18.67 (11.46–28.93)	Ref	
Unmarried:	26	31.54 (16.84–46.24)	13	50.00 (32.06–67.94)	10	38.46 (22.43–57.47)	6	23.08 (11.03–42.05)	0.87 (0.43–1.76)	0.70
Separated:	30	25.96 (15.40–36.52)	14	46.67 (30.23–63.86)	9	30.00 (16.66–47.88)	5	16.67 (7.34–33.56)	0.86 (0.40–1.87)	0.71
Unknown:	9	28.00 (15.45–40.55)	–		–		–		–	–
Insurance:										0.002
Yes:	105	28.06 (22.56–33.56)	63	60.00 (50.44–68.86)	30	28.57 (20.81–37.85)	18	17.14 (11.13–25.48)	Ref	
No:	8	10.25 (0.00–22.04)	1	12.50 (2.24–47.09)	1	12.50 (2.24–47.09)	0	–	1.96 (1.18–3.27)	0.009
unknown	27	48.54 (27.33–69.76)							–	–
Tumor grade:										0.00
I:	56	47.57 (37.67–57.48)	50	89.29 (78.53–95.00)	28	50.00 (37.33–62.67)	16	28.57 (18.42–41.48)	Ref	
II:	25	41.84 (27.99–55.68)	18	72.00 (52.42–85.72)	11	44.60 (26.67–62.93)	8	32.00 (17.21–51.59)	0.16 (0.08–0.32)	0.00
III:	47	9.57 (4.66–14.48)	11	23.40 (13.60–37.22)	2	4.26 (1.17–14.25)	1	2.13 (0.38–11.11)	0.20 (0.10–0.41)	0.29
IV:	12	5.58 (0.00–12.36)	1	8.33 (1.49–35.39)	0	–	0	–	0.71 (0.37–1.34)	0.00
Unknown:	0									
Tumor size:										0.44
< 3 cm:	22	35.14 (16.93–53.34)	13	59.09 (38.73–76.74)	7	31.82 (16.36–52.68)	5	22.73 (10.12–43.44)	Ref	
3-5 cm:	18	32.39 (16.70–48.09)	11	61.11 (38.62–79.69)	6	33.33 (16.28–56.25)	4	22.22 (9.00–45.21)	0.61 (0.35–1.08)	0.09
5-10 cm:	32	32.22 (19.53–44.91)	18	56.25 (39.33–71.83)	12	37.50 (22.93–54.75)	7	21.88 (11.02–38.75)	0.72 (0.41–1.29)	0.28
> 10 cm:	34	33.98 (21.33–46.62)	20	58.82 (42.22–73.63)	11	32.35 (19.13–49.16)	7	20.59 (10.35–36.80)	0.73 (0.45–1.20)	0.21
Unknown:	34	21.88 (13.10–30.65)							–	–
Lymph node invasion:										0.007
No:	115	32.72 (26.30–39.30)	71	61.74 (52.61–70.11)	38	33.04 (25.12–42.07)	23	20.00 (13.71–28.23)	Ref	
Yes:	12	13.00 (0.54–25.46)	5	41.67 (19.33–68.05)	3	25.00 (8.89–53.23)	1	8.33 (1.49–35.39)	0.89 (0.49–1.63)	0.09
Unknown:	13	29.58 (3.69–55.48)							–	–
Total tumor number:										0.27
1:	92	33.46 (25.89–41.04)	54	58.70 (48.48–68.21)	30	32.61 (23.89–42.72)	20	21.74 (14.54–31.21)	Ref	
2:	36	22.19 (14.10–30.29)	20	55.56 (39.58–70.46)	9	25.00 (13.75–41.07)	3	8.33 (2.87–21.83)	2.08 (0.29–14.99)	0.47
3:	11	32.18 (2.96–61.41)	5	45.45 (21.27–71.99)	2	18.18 (5.14–47.70)	1	9.09 (1.62–37.74)	2.94 (0.40–21.62)	0.29
4:	1	78.00	1	–	1	–	1	–	2.13 (0.27–16.66)	0.47
AFP:										0.12
Elevated:	12	21.67 (3.82–39.52)	4	33.33 (13.81–60.94)	3	25.00 (8.89–53.23)	3	25.00 (8.89–53.23)	Ref	
Normal:	35	40.20 (25.43–54.97)	20	57.14 (40.86–72.02)	14	40.00 (25.55–56.43)	10	28.57 (16.33–45.05)	1.29 (0.70–2.37)	0.41
Unknown:	93	28.24 (21.86–34.63)							–	–
Fibrosis Score										0.78
Fibrosis score 0–4:	13	25.62 (10.57–40.66)	7	53.85 (29.14–76.79)	3	23.08 (8.18–50.26)	2	15.38 (4.33–42.23)	Ref	
Fibrosis score 5–6:	2	39.00 (0.00–115.44)	1	–	1	–	1	–	1.21 (0.68–2.15)	0.51
Unknown:	125	31.12 (24.76–37.49)							–	–
Surgery										0.00
Yes:	45	54.16 (42.34–65.98)	40	88.89 (76.50–95.16)	26	57.78 (43.30–71.03)	19	42.22 (28.97–56.70)	Ref	
No:	95	19.25 (14.03–24.48)	39	41.05 (31.70–51.10)	16	16.84 (10.64–25.62)	7	7.37 (3.61–14.44)	0.38 (0.26–0.56)	0.00
Chemotherapy:										0.34
Yes:	34	25.68 (15.71–35.64)	18	52.94 (36.74–68.55)	8	23.53 (12.44–40.00)	4	11.76 (4.67–26.62)	Ref	
No/unknown:	106	32.43 (25.32–39.54)	61	57.55 (48.04–66.53)	34	32.08 (23.95–41.45)	22	20.75 (14.12–29.43)	1.21 (0.82–1.79)	0.35
Radiation:										0.97
Yes:	4	29.75 (18.09–41.41)	3	75.00 (30.06–95.44)	2	50.00 (15.00–85.00)	0	–	Ref	
No/unknown:	136	30.75 (24.69–36.82)	75	55.15 (46.76–63.25)	40	29.41 (22.40–37.55)	26	19.12 (13.39–26.54)	1.02 (0.37–2.77)	0.97

Tumor grade: I: Well differentiated, II: moderately differentiated, III: poorly differentiated, IV: undifferentiated;

**Table 2:** 1-year, 3-year, 5-year survival rate in four cohorts

Items	Survival time	Survival rate		
	Mean survival time [95% CI] (months)	1-year survival (95% CI)	3-year survival (95% CI)	5-year survival (95% CI)
Primary hepatic neuroendocrine tumors (PH-NETs):	30.64 (24.75–36.53)	56.43 (48.15–64.36)	30.00 (23.03–38.04)	18.57 (13.00–25.82)
Hepatocellular carcinoma (HCC in situ):	25.11 (20.20–30.02)	55.00 (46.74–63.00)	21.43 (15.44–28.94)	10.71 (6.60–16.93)
Gastrointestinal neuroendocrine tumors in situ (GI-NETs in situ):	41.62 (35.32–47.92)	74.29 (66.47–80.81)	42.86 (34.96–51.14)	27.86 (21.10–35.80)
Gastrointestinal neuroendocrine tumors with liver metastasis (GI-NETs-LM):	24.81 (21.15–36.60)	64.75 (56.51–72.20)	30.94 (23.85–39.05)	8. 63 (5.01–14.48)

Most included patients were aged> 25 years, only 1 patient was aged within 0–24 years. 39.28% of the patients are male and 75.00% have insurance. Among all, 115 patients have no record of lymph nodes invasion, 45 patients have “surgery” and 34 patients have “chemotherapy” (Table 1).

Univariate analysis was conducted and the overall 1-year, 3-year, and 5-year survival rate results were shown respectively (Table 1). For the univariate analysis, older age, lack of insurance, advanced tumor grade, lymph nodes invasion were the risk factors associated with unfavorable prognosis. Based on the result of age, the 1-year, 3-year, 5-year survival rates for patients with the age of 25–49 and 50–74 were 78.57, 53.57, 32.14 and 52.94%, 32.35, 22.06%, respectively, compared with merely 46.51, 11.63 and 4.65% in patients aged over 75 (*p* = 0.002, Table 1). Similarly, the 1-year, 3-year and 5-year survival rates for patients with tumor grade I and II were 89.29, 50.00, 28.57 and 64.7%, 54.6, 19.9%, respectively. When compared with the rate of grade III (23.40, 4.26 and 2.13%), the difference was significant (*p <* 0.001) and grade IV patients suffered an even lower survival chance. Predictably, patients with lymph nodes invasion demonstrated significantly longer mean survival time with 32.72 months compared with 13.00 months (*p* = 0.007). Additionally, better insurance status was associated with advantage clinical outcomes (*p* = 0.002, Table 1). In contrast, none of the sex difference, tumor size, total tumor number, AFP or fibrosis score have significant influence on patients’ survival (Table 1).

As for the management options, three treatment measures were included, only surgical had the capacity to effectively boost overall survival. The mean survival time was 54.16 months for patients received surgery, which significantly exceeded 19.25 months of those who did not (*p <* 0.001). Surprisingly, no significant relationship between chemotherapy and survival benefits was revealed (Table 1).

It is worth mentioning that, 5 were treated with liver transplantation among 45 patients received surgery, their survival time were 90, 93, 81 and 26 months. Detailed information was in supplementary Table 1.

### PH-NETs patients’ survival comparison with three cohorts from PSM analysis

In an attempt to have a comprehensive understanding of the PH-NETs prognosis, we derived three cohorts from the SEER database by using propensity-score matching (PSM) analysis at a 1: 1 ratio. As described in supplementary Table 2, we selected age, race, sex, marital status, insurance status, tumor grade, tumor size, lymph node metastasis, total tumor number, surgery, chemotherapy and radiation in the match criteria.

**Fig. 1:**
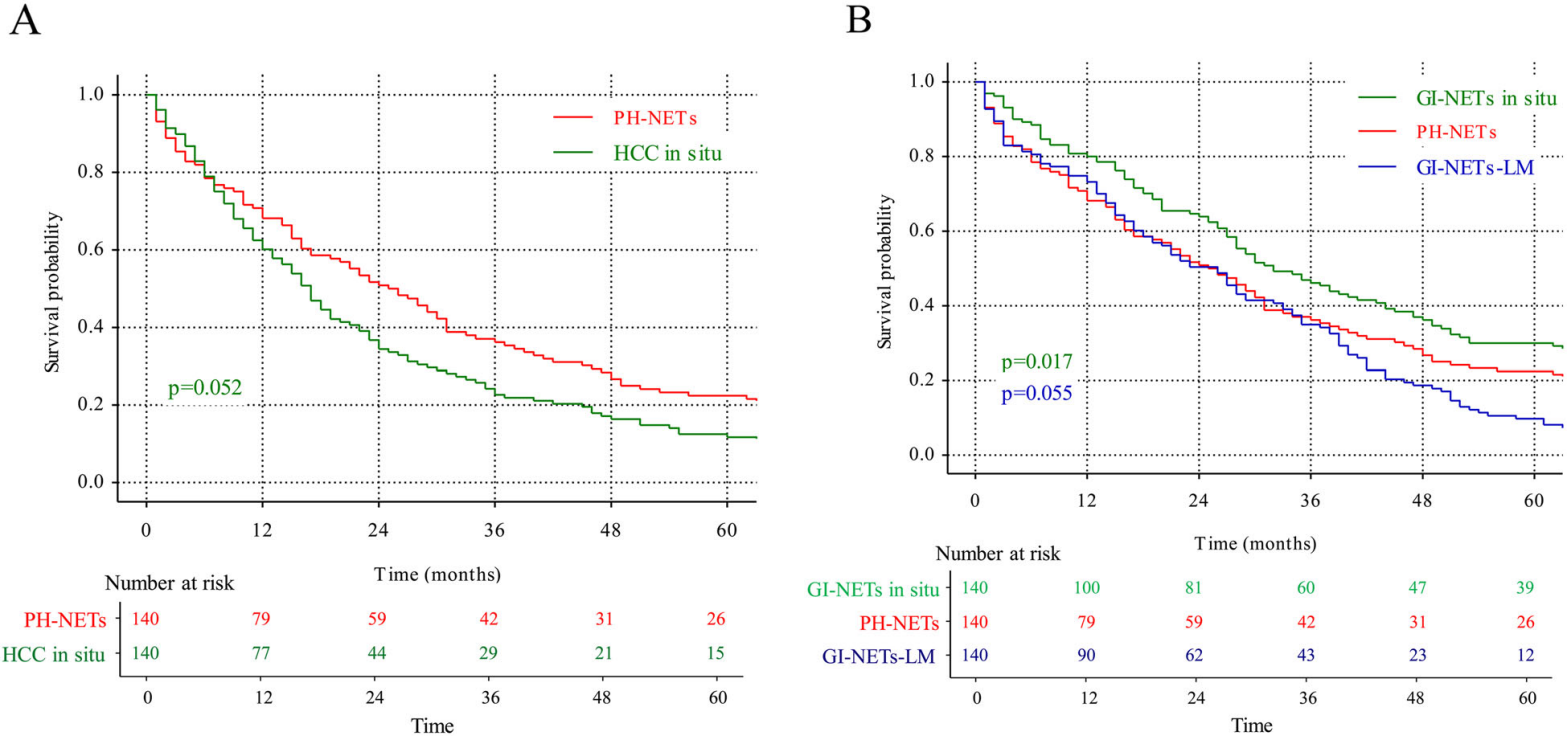
Survival curves of PH-NETs and three cohorts from PSM analysis. **a**: Survival comparison between PH-NETs and HCC in situ. **b**: Survival comparison between PH-NETs and GI-NETs in situ; Survival comparison between PH-NETs and GI-NETs-LM

The first was a HCC in situ cohort, 140 patients who were diagnosed HCC without metastasis were selected. The patients’ characteristics were revealed in supplementary Table 2. Concerning the prognostic outcomes between PH-NETs and matched HCC in situ patients, survival rate was 56.43% vs 55.00% in 1-year survival, 30.00% vs 21.43% in 3-year survival, 18.57% vs 10.71% in 5-year survival, demonstrating a better prognosis in PH-NENs patients (Table 2). However, following log-rank analysis failed to detect significant difference (Fig. 1a, *p* = 0.052).

Similarly, because liver is the most frequent metastatic site for GI-NETs and PH-NETs diagnosis requires exclusion of extrahepatic primary sites. We separately constructed two GI-NETs cohorts which were GI-NETs in situ and GI-NETs with liver metastasis (GI-NETs-LM). Each included 140 patients by using propensity-score matching (PSM) analysis. Patients’ characteristics were also showed in supplementary Table 2. As for GI-NENs in situ, result demonstrated there was a significant difference between GI-NENs in situ and PH-NETs patients. GI-NENs in situ patients had significant better prognosis outcomes. Survival curve was drew and log-rank analysis was performed (Fig. 1b, *p* = 0.017).

Contrast with GI-NETs in situ cohort, there was a dramatic decreased long-term survival chance in GI-NETs-LM. In the comparison between GI-NETs-LM and PH-NETs, the two cohorts have almost same 3-year survival rate (30.94% vs 30.00% in Table 2). In a follow-up time less than 3 years, GI-NETs-LM enjoyed a better prognosis than PH-NETs (56.43% vs 64.75% in 1-year survival). However, the survival of GI-NETs-LM declined rapidly after 3 years (8.63% vs 18.57% in 5-year survival) although the log-rank analysis showed no significant difference in overall survival (Fig. 1b, *p* = 0.055).

### Multivariate cox analyses for PH-NETs prognostic outcome

In univariate analysis, a total of 5 variables incorporated significant influence in prognostic outcome (Table 1). The 5 factors were further included in the multivariate Cox analysis and 2 variables were left as revealed in Table 3. Advanced tumor grade acts as the exclusive adverse prognostic factors for PH-NETs (HR = 2.03, 95%CI = 1.6–2.48, *p <* 0.00). Conversely, surgery was proved to be the most effective treatment measure and possessed the highest hazard ratio (HR = 2.72, 95%CI = 1.84–4.00, *p <* 0.00). Regression coefficient for tumor grade and surgery was also calculated.

**Table 3:** Multivariate Cox analyses in PH-NETs prognostic outcome

Variables	Multivariate analysis		Regression coefficient
	HR [95% CI]	*P*-value	
Tumor grade:	2.03 (1.66–2.48)	0.00	0.708
Surgery:	2.72 (1.84–4.00)	0.00	−0.998

**Fig. 2:**
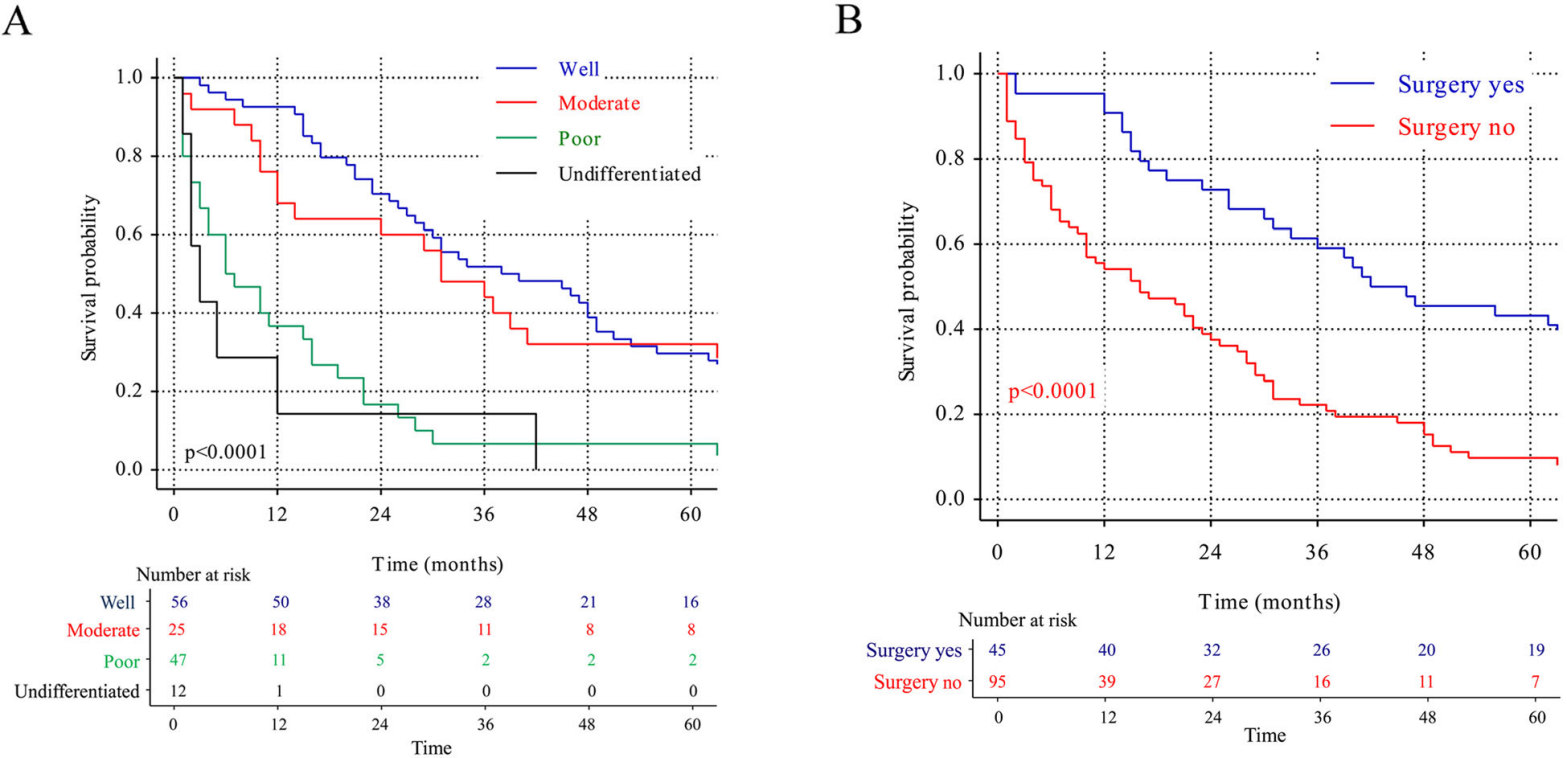
Survival for patients with PH-NETs stratified by (**a**) tumor grade; and (**b**) surgery

To better display the results in multivariate Cox analysis, survival curves were created for the 2 identified prognostic factors (Fig. 2). Tumor grade and surgery survival curves were shown in Fig. 2a and b.

### Prognostic nomogram construction for PH-NETs patients

**Fig. 3:**
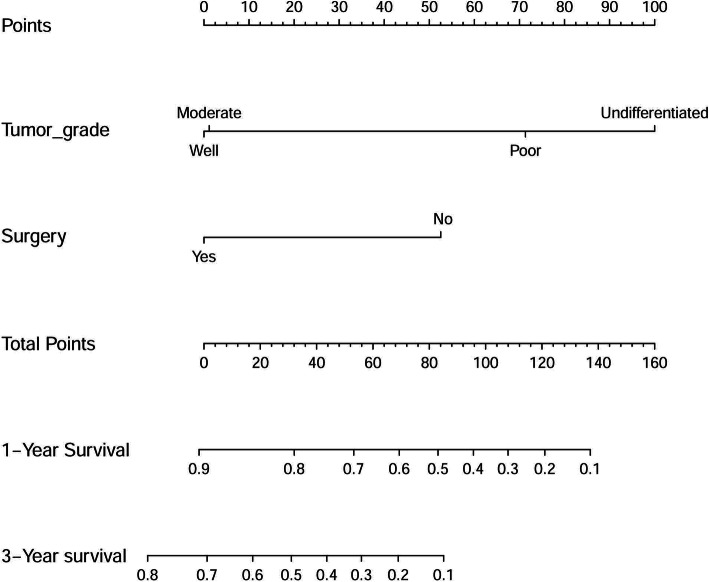
Prognostic nomogram estimated by clinical features for the overall 1-year, 3-year, and 5-year survival rate in PH-NETs patients

In order to predict the survival of PH-NETs patients, a nomogram was further constructed by incorporating the 2 filtered independent indicators identified by the multivariate analyses (Fig. 3).

**Fig. 4:**
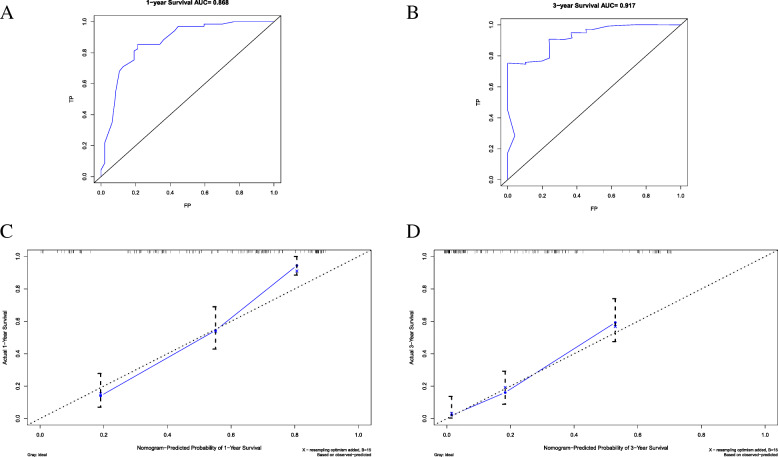
The receiver operating characteristic (ROC) curve for prognosis prediction precision of the nomogram model at (**a**) 1-year and (**b**) 3-year. The calibration plots for predicting PH-NETs patients’ survival at (**c**) 1-year and (**d**) 3-year

To measure the nomogram accuracy, a C-index of 0.79 (95%CI = 0.75–0.83) was concluded. Furthermore, the ROC models regarding the predictive ability of 1-year and 3-year survival were constructed (Fig. 4a and b) and the resulting AUC values were 0.868 and 0.917, respectively. In addition, the calibration curves for predicting patients’ 1-year and 3-year survival were also drew and demonstrated good prediction ability (Fig. 4c and d).

## Discussion

PH-NETs are very rare tumors of liver. Initially, 635 patients were identified in the SEER database from 1975 to 2016. Patients with insufficient pathological diagnosis information were eliminated. In view of the difficulty in distinguishing primary and metastatic liver neuroendocrine tumors, so we excluded patients with any distant metastasis from the cohort. Finally, a total of 140 patients with PH-NETs were included in the present study.

More than a rare entity, its diagnosis is difficult. Preoperative fine needle biopsy is strongly recommended [26, 27]. However, it must be pointed out that an apparent PH-NET may be metastatic disease, despite after extensive assessment such as endoscopy, colonoscopy and even capsule gastroscopy, the primary tumor remains obscure. Since the SEER database we used traced back to 1975, occult primary tumors detection would be particularly challenging due to lack of high sensible imaging technology at that time. PET-CT (positron emission computed tomography) has unique advantage in locating tumor metastasis. However, classical PET with fluorodeoxyglucose did not prove to be advantageous in NETs imaging [28]. In order to improve diagnostic accuracy of primary site in NETs from unknown origin, 68Ga-DOTApeptides-PET/CT was increasingly used without recorded adverse effects and therefore was first suggested in patients with diagnostic difficulty [29]. With the help of serotonin precursor 11C-5 hydroxy tryptophan, a newly-developed PET tracer, the identification accuracy of the primary NENs was promoted to 84% of all cases [30, 31]. Octreoscan® scintigraphy, which was also a specific imaging tool in NETs diagnosis, was also found to surpass routine CT and MRI in detecting primary NETs and distant metastasis [32].

For decades NETs were often assumed to be benign and excluded from cancer registries, making rigorous and accurate epidemiologic studies difficult [33]. The overall prognosis of PH-NETs was even more obscure owing to its rarity although case reports implicated better prognosis than common-type hepatic carcinomas like HCC [34–36]. To our knowledge, this is the first study provided thorough prognosis profile of PH-NETs with the comparison to HCC and GI-NENs with/without liver metastasis. As reported, although with a *p* value slight over 0.05, our study proved that PH-NETs have a better prognosis than HCC (Table 2). According to the current WHO International Classification of Diseases for Oncology, version 3 (ICD-O.3), PH-NETs is recognized as one of the subtypes of NETs [37]. Therefore, we decided to compare the prognosis of PH-NETs with NETs from other sites, in this we selected GI-NETs which is the most common NETs subtype. GI-NETs cohort was further divided into GI-NETs in situ cohort and GI-NETs-LM cohort because distant metastasis was excluded from PH-NETs. Both GI-NETs in situ cohort and GI-NETs-LM cohort has included 140 patients by using PSM analysis. As a result, the prognosis of GI-NETs in situ is much better than PH-NETs with *p* = 0.017. As for GI-NETs-LM, the short-term and long-term survival rate showed different result when compared to PH-NETs. The survival rate of GI-NETs-LM quickly declined and long-term survival is much lower than PH-NETs. The underlying meaning behind this phenomenon highlights that liver involvement is an essential prognostic factor in NETs patients, no matter it is primary or metastatic. Therefore, early detection of distant metastasis may be of great essential to achieve better outcomes in NETs patients.

Cox multivariate analysis indicated that tumor grade was regarded as the most deteriorating factor and increased the risk of death of PH-NETs patients. As a matter of fact, a prior study in Turkey concluded that grade rather than stage was the most important prognostic factors in overall NETs [38]. When deeply analyzed, the survival rates between grade I and II were almost the same (89.29% vs 72.00% in 1-year, 50.00% vs 44.60% in 3-year, 28.57% vs 32.00% in 5-year, Table 1, Fig. 2a). The long-term survival in grade I/II patients reached nearly 30% indicating a favorable prognosis. Nevertheless, with tumor grade upgraded to III/IV, the survival rate declined in a surprising rate and became even worse than HCC (23.40% vs 55.00% in 1-year, 4.26% vs 21.43% in 3-year). Furthermore, the long-term survival (5-year) was extremely low, close to 2%. In accordance to recent updates on grading and classification of neuroendocrine tumors, ki-67 labeling rate is the best indicator for tumor grading and > 20% means tumor grade III or more [39]. Thus, preoperative biopsy and tumor grade staining was strongly suggested in PH-NETs diagnosis.

In literature, the most recommended treatment of PH-NETs is surgery [14, 18, 36]. In our study, there was significant discrepancy in prognosis between surgery and non-surgery patients. The 5-year survival rate for patients would reach up to 42.22% if patients undergo surgery timely. With regard to other therapies, such as chemotherapy, hepatic artery chemoembolization (TACE), somatostatin hormone therapy or its analogs, they are mainly used in unresectable lesions or patients with distant metastasis [14, 40–42]. The efficacy of chemotherapy still remains controversial with some patients failed to respond to systemic chemotherapy [43]. Our study revealed that chemotherapy failed to be considered as prognostic factors and thus were not sufficient to accomplish long-term survival. TACE has been reported to cause tumor bulk reduction when used alone or integrated with surgery [26, 44]. Because TACE was not recorded in SEER database, data paucity makes it difficult to evaluate its effectiveness. Hormone therapy is indicated in carcinoids with NETs symptoms, however, this therapy might only exert cytostatic effects [45]. No evidence is available as to the control of tumor growth or disease progression, further studies are needed to evaluate this effect [46].

Notably, in terms of surgical strategies, liver transplantation has appeared to be the best therapeutic alternative for unresectable lesion. In our study, 4 patients had liver transplantation, 2 of them were grade I/II and survival time were 90, 93 months. The other 2 grade III patients survived 81 and 26 months respectively. According to a study in United Kingdom, the outcome of liver transplantation in two patients was favorable, with no disease recurrence at 45 and 95 months [47, 48]. A review summarizing the use of liver transplantation in metastatic liver NETs insights that, the ideal candidate criteria should include: (1) No extrahepatic metastases; (2) Less than 50% of liver involvement; (3) ki-67 < 5–10%; (4) Good tumor grade; (5) Age < 50–60 years (minor criteria); (6) Stable disease> 6 months [49]. These criteria were also applicable to PH-NETs with little revision. Moreover, a retrospective designed, single-center study containing 22 PH-NETs patients concluded that patients exhibiting lower proliferative grade (grade I/II) would especially expect longtime survival after surgical management [50]. This evidence suggested the importance of radical surgical approach including liver transplantation in the management of PH-NETs or even in the treatment of metastatic liver NETs with resectable primary site.

In our study, a convenient nomogram containing tumor grade and surgery was constructed for predicting PH-NETs patients’ survival. To our knowledge, this is the first nomogram in this field based on the SEER database with long-term follow-up, thus assisting surgeons and patients to produce individualized survival prediction via the scoring system.

Nevertheless, it should be mentioned that this study had some limitations. Some are intrinsic to any retrospective analyses of SEER database [51]. For example, the SEER database does not provide the data of TACE, which is a widely used therapy in liver neoplasms. The SEER database also failed to confer the comprehensive factors concerning the severity of PH-NETs patients, such as Child-Pugh Score, large vascular invasion, et al. Furthermore, SEER database merely collects cancer information among American population, and PH-NETs is coincidentally reported a predominance in Asian countries [52, 53]. Besides these, a specific concern of this study would be that we excluded all cases with metastases. Part of the reason is that PH-NETs has not been recognized as an independent tumor type in liver, so PH-NETs with distant metastasis would more likely be considered as metastatic liver NETs. What’s more, in initial analyses of the 290 patients after removal of insufficient pathological diagnosis, multivariate Cox analyses still discovers grade and surgery as influencing factors with high HR, with age and sex also showed significant difference (data not shown). Considering this, 140 cases were included for more accurate conclusion.

## Conclusions

Collectively, our study first comprehensively described the prognosis of PH-NETs, a rare entity by using the SEER database. We revealed two significant factors influencing PH-NETs prognosis. To be specific, tumor grade III/IV or ki-67 > 20% indicated unfavorable prognostic for PH-NETs patients. Surgery was recommended as a curative treatment to confer a significant survival advantage. In case of unresectable tumor, liver transplantation or intervention therapy such as TACE was suggested. On the basis of this, we further formulated the first nomogram to predict long-term survival, which may facilitate disease course prediction and optimize clinical management strategies in such patient population.

## Supplementary Information

**Additional file 1: Supplementary Table 1.** Patient characteristics for PH-NETs and HCC after PSM analysis.

**Additional file 2: Supplementary Table 2.** Characteristics of patient undergo liver transplantation.

## Data Availability

The SEER program is publicly accessible so the data was publicly available for all researchers. The link to SEER database is https://seer.cancer.gov/.
