# Supplementary material for: Development of a nomogram model to predict survival outcomes in patients with primary hepatic neuroendocrine tumors based on SEER database
Source: BMC Cancer. 2021 May 18;21:567. doi: 10.1186/s12885-021-08337-y (PMC8130428; doi:10.1186/s12885-021-08337-y)
Supplement: Supplementary file 1 — Additional file 1: Supplementary Table 1. Patient characteristics for PH-NETs and HCC after PSM analysis. [file 12885_2021_8337_MOESM1_ESM.docx]

Supplementary table 1: Patient characteristics for PH-NETs and HCC after PSM analysis

| Characteristics : | Primary hepatic neuroendocrine tumors (PH-NETs) | Hepatocellular carcinoma (HCC in situ) | Gastrointestinal neuroendocrine tumors in situ (GI-NETs in situ): | Gastrointestinal neuroendocrine tumors with liver metastasis (GI-NETs-LM): |
| --- | --- | --- | --- | --- |
| Total number: | 140 | 140 | 140 | 140 |
| Age (years): |  |  |  |  |
| 00-24: | 1 | 1 | 1 | 0 |
| 25-49: | 28 | 10 | 16 | 30 |
| 50-74: | 68 | 95 | 104 | 81 |
| >75: | 43 | 34 | 19 | 29 |
| Race: |  |  |  |  |
| Black: | 24 | 13 | 37 | 16 |
| Other: | 9 | 29 | 11 | 8 |
| White: | 107 | 97 | 84 | 116 |
| Unknown: | 0 | 1 | 8 | 0 |
| Sex: |  |  |  |  |
| Female: | 85 | 68 | 80 | 64 |
| Male: | 55 | 72 | 60 | 76 |
| Marital status: |  |  |  |  |
| Married: | 75 | 78 | 65 | 72 |
| Unmarried: | 26 | 16 | 26 | 19 |
| Separated: | 30 | 33 | 25 | 36 |
| Unknown： | 9 | 13 | 27 | 13 |
| Insurance: |  |  |  |  |
| Yes: | 105 | 120 | 105 | 130 |
| No: | 8 | 4 | 7 | 5 |
| Unknown: | 27 | 16 | 28 | 5 |
| Tumor grade: |  |  |  |  |
| I: | 56 | 50 | 67 | 63 |
| II: | 25 | 26 | 18 | 26 |
| III: | 47 | 5 | 24 | 27 |
| IV: | 12 | 0 | 2 | 4 |
| Unknown： | 0 | 59 | 29 | 20 |
| Tumor size: |  |  |  |  |
| <3cm: | 22 | 34 | 46 | 37 |
| 3-5cm: | 18 | 26 | 20 | 15 |
| 5-10cm: | 32 | 39 | 11 | 32 |
| >10cm: | 34 | 15 | 11 | 5 |
| Unknown: | 34 | 26 | 52 | 51 |
| Lymph node invasion: |  |  |  |  |
| No: | 115 | 128 | 92 | 108 |
| Yes: | 12 | 8 | 39 | 27 |
| Unknown: | 13 | 4 | 9 | 5 |
| Total tumor number: |  |  |  |  |
| 1: | 92 | 115 | 85 | 102 |
| 2: | 36 | 16 | 41 | 29 |
| 3: | 11 | 7 | 9 | 6 |
| 4: | 1 | 2 | 5 | 3 |
| Surgery: |  |  |  |  |
| Yes: | 45 | 34 | 60 | 47 |
| No: | 95 | 106 | 80 | 93 |
| Chemotherapy: |  |  |  |  |
| Yes: | 34 | 47 | 20 | 25 |
| No/unknown: | 106 | 93 | 120 | 115 |
| Radiation: |  |  |  |  |
| Yes: | 4 | 30 | 4 | 12 |
| No/unknown: | 136 | 110 | 136 | 128 |

Tumor grade: I: Well differentiated, II: moderately differentiated, III: poorly differentiated, IV: undifferentiated;
