# Supplementary material for: Development of a nomogram model to predict survival outcomes in patients with primary hepatic neuroendocrine tumors based on SEER database
Source: BMC Cancer. 2021 May 18;21:567. doi: 10.1186/s12885-021-08337-y (PMC8130428; doi:10.1186/s12885-021-08337-y)
Supplement: Supplementary file 2 — Additional file 2: Supplementary Table 2. Characteristics of patient undergo liver transplantation. [file 12885_2021_8337_MOESM2_ESM.docx]

Supplementary table 2 Characteristics of patient undergo liver transplantation

| Patient number | Age (years) | Race | Sex | Year of diagnosis | Insurance Recode | Marital status | Tumor grade | Tumor size (mms) | Lymph node invasion | Total tumor number | Chemotherapy | Radiation | Survival time (months) |
| --- | --- | --- | --- | --- | --- | --- | --- | --- | --- | --- | --- | --- | --- |
| 1 | 45-49 | B | F | 2006 | NA | Married | I | 250 | No | 1 | No | None | 90 |
| 2 | 25-29 | W | F | 2009 | Insured | Married | II | 200 | No | 1 | No | None | 93 |
| 3 | 55-59 | W | M | 2010 | Insured | Divorced | III | 68 | NA | 1 | No | None | 81 |
| 4 | 65-69 | B | F | 2014 | Insured | Married | III | 152 | No | 2 | No | None | 26 |

Sex: W: white; B: black;

NA: not available;

Tumor grade: I: Well differentiated, II: moderately differentiated, III: poorly differentiated, IV: undifferentiated;
